# Chicago Society of Physicians and Surgeons

**Published:** 1873-09-01

**Authors:** 


### Medical Examiner.

*A Semi-Monthly Journal of Medical Sciences.*

EDITED BY

N. S. DAVIS, M. D., AND F. H. DAVIS, M. D.

Chicago, Sept. 1st, 1873.

### EDITORIAL.

### CHICAGO SOCIETY OF PHYSICIANS AND SURGEONS.

SPECIAL MEETING, MONDAY EVENING, AUGUST 18TH, 1873.

The meeting was called to continue the discussion on Cholera. Dr. Owens in the chair.

Dr. Simons made the following verbal report: There had been ten deaths from cholera since the last meeting. The Cholera Hospital, situated near Burnside st., between 33d and 35th sts., was opened Tuesday, Aug. 12th. A number of patients had been received; and those taken there, if not too far advanced, did much better than if they had remained at home. The disinfectants used were carbolic acid, sulphate of iron, and chloride of lime. There were four cases in the hospital, and all but one were doing well. Two of the nurses were taken with the premonitory symptoms, but soon recovered. Little attention is paid by the people of that section to the circulars issued by the Board of Health; and when they call a physician, even in collapse, they say they have nothing but a diarrhœa. One-third of the inhabitants have left the infected region. In spite of all that could be said, the Germans would not destroy their feather beds on which cholera patients had died, and when they move away carry the beds and contagion with them. There had appeared to be no abatement in the endemic since the introduction of lake water into the district. Some families would persist in using the surface well water, wholly or in part, while others had been using only the hydrant lake water. The latter families, however, who had used only pure water, seemed to suffer equally with their neighbors.

Dr. Bert next made some remarks regarding the treatment pursued with cholera patients in the hospital at Hamburg, during his residence there. He called especial attention to the treatment by warm baths during the stage of collapse. Under that course of treatment, he claimed that the deaths averaged only 47 per cent. The warm bath, he stated, had also proved remarkably efficient in cases of pyæmia resulting from gunshot wounds.

The Secretary, Dr. Hyde, stated that a specimen of the cholera dejections had been submitted to Dr. I. N. Danforth for examination under the microscope, but that Dr. Danforth would not be able to present a re-report until the next meeting.

Dr. Wood had obtained some of the water from a filter well at No. 947 Burnside street, south of city limits. The well was ten feet below the surface, and contained surface water. The water was filtered through two layers of sand and one of charcoal. Upon applying the permanganate of potassa test, he found that the water contained 91.279 grs. of oxidizable organic matter to the gallon, which consisted mostly of broken down vegetable matter, as was demonstrated by microscopic examination.

Dr. C. G. Smith had seen no cases of cholera. He mentioned, however, a case of acute dysentery, which terminated fatally in thirty-six hours from the onset of the attack. Death resulted, apparently, from exhaustion, occasioned by the excessive hæmorrhage.

In reply to a question from Dr. Smith, Dr. Simons stated that a large proportion of the cases falling under his care did have more or less painless diarrhœa, for a variable time preceding the onset of the more violent symptoms. When prompt measures were at once taken, during this stage, there was no difficulty in controlling the disease.

On motion, two committees were appointed, consisting of five members each, the one to prepare as complete a history as possible of the origin and spread of this endemic, prevailing in the southwest section of the city; and the other to report on the best treatment to be pursued in the different stages of cholera; after which the Society adjourned to the next regular meeting, Monday evening, August 25th.

There was a large attendance of members, as well as many visitors present, and much interest was manifested by all in the discussions.

Dr. J. R. Moore, of Stoughton, Wis., reports a case of sporadic cholera in his practice. The patient was taken with severe vomiting, purging and cramps, Aug. 2d, at 11 P.M., and died in collapse, Aug. 4th, at 9 P.M.

